# Anesthetics to Prevent Lung Injury in Cardiac Surgery (APLICS): a protocol for a randomized controlled trial

**DOI:** 10.1186/s13063-019-3400-x

**Published:** 2019-05-31

**Authors:** Brian O’Gara, Balachundhar Subramaniam, Shahzad Shaefi, Ariel Mueller, Valerie Banner-Goodspeed, Daniel Talmor

**Affiliations:** Department of Anesthesia, Critical Care and Pain Medicine, Beth Israel Deaconess Medical Center, Harvard Medical School, 330 Brookline Ave, Boston, MA 02215 USA

**Keywords:** Inflammatory lung injury, Volatile anesthetics, Cardiac surgery, Postoperative pulmonary complications, TNFα

## Abstract

**Background:**

Patients undergoing cardiac surgery with cardiopulmonary bypass are at an increased risk of developing postoperative pulmonary complications, potentially leading to excess morbidity and mortality. It is likely that pulmonary ischemia-reperfusion (IR) injury during cardiopulmonary bypass is a major contributor to perioperative lung injury. Therefore, interventions that can minimize IR injury would be valuable in reducing the excess burden of this potentially preventable disease process. Volatile anesthetics including sevoflurane have been shown in both preclinical and human trials to effectively limit pulmonary inflammation in a number of settings including ischemia-reperfusion injury. However, this finding has not yet been demonstrated in the cardiac surgery population. The Anesthetics to Prevent Lung Injury in Cardiac Surgery (APLICS) trial is a randomized controlled trial (RCT) investigating whether sevoflurane anesthetic maintenance can modulate pulmonary inflammation occurring during cardiac surgery with cardiopulmonary bypass and whether this potential effect can translate to a reduction in postoperative pulmonary complications.

**Methods:**

APLICS is a prospective RCT of adult cardiac surgical patients. Participants will be randomized to receive intraoperative anesthetic maintenance with either sevoflurane or propofol. Patients in both groups will be ventilated according to protocols intended to minimize the influences of ventilator-induced lung injury and hyperoxia. Bronchoalveolar lavage (BAL) and blood sampling will take place after anesthetic induction and 2–4 h after pulmonary reperfusion. The primary outcome is a difference between groups in the degree of post-bypass lung inflammation, defined by BAL concentrations of TNFα. Secondary outcomes will include differences in additional relevant BAL and systemic inflammatory markers and the incidence of postoperative pulmonary complications.

**Discussion:**

APLICS investigates whether anesthetic choice can influence lung inflammation and pulmonary outcomes following cardiac surgery with cardiopulmonary bypass. A positive result from this trial would add to the growing body of evidence describing the lung protective properties of the volatile anesthetics and potentially reduce unnecessary morbidity for cardiac surgery patients.

**Trial registration:**

ClinicalTrials.gov, NCT02918877. Registered on 29 September 2016.

**Electronic supplementary material:**

The online version of this article (10.1186/s13063-019-3400-x) contains supplementary material, which is available to authorized users.

## Background

Patients undergoing cardiac surgery with cardiopulmonary bypass (CPB) are at an increased risk for postoperative pulmonary complications (PPCs), with recent estimates of the incidence as high as 50% [1, 2]. Overt respiratory failure and acute respiratory distress syndrome (ARDS) can occur in as many as 10% of patients, leading to mortality rates up to 40 times higher than patients without these conditions [3]. Pulmonary ischemia-reperfusion (IR) injury during CPB has been identified as a major contributor to perioperative lung injury, but to date strategies to mitigate such injury have failed to demonstrate a consistent benefit [4, 5]. Volatile anesthetics have been shown to protect the lung from various etiologies of inflammatory lung injury in both preclinical models and in humans [6–9]. Proposed mechanisms for this observed effect include a reduction in the release of inflammatory mediators from pulmonary neutrophils and macrophages and preservation of alveolar endothelial integrity [10]. Although the volatile anesthetic sevoflurane has been shown to prevent myocardial injury from IR after cardiac surgery, there has been a lack of data showing whether its use in this population can result in a similar level of protection from IR lung injury [11]. Given the excess morbidity and mortality associated with PPCs in this population, investigation into whether the use of volatile anesthetics such as sevoflurane can reduce lung injury after CPB could impact the outcomes of thousands of cardiac surgical patients a year [12].

## Methods and design

### Study design

**Fig. 1:**
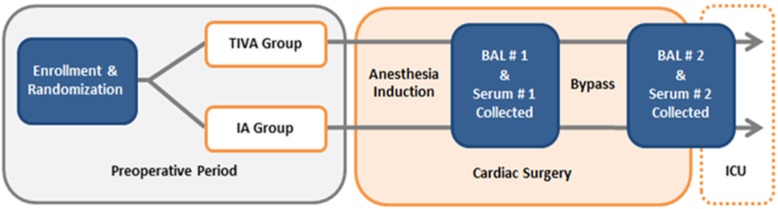
APLICS study schema. TIVA total intravenous anesthesia (propofol), IA inhaled anesthetic (sevoflurane), ICU intensive care unit

The Anesthetics to Prevent Lung Injury in Cardiac Surgery (APLICS) trial is a randomized, controlled, single-center clinical trial of adult cardiac surgical patients undergoing CPB. Individuals are randomized to receive intraoperative anesthetic maintenance with either sevoflurane or propofol. Lung inflammation will be evaluated by analyzing differences in the pre- and post-bypass pulmonary tumor necrosis factor alpha (TNFα) between groups, obtained via a bronchoalveolar lavage (BAL). Secondary outcomes include differences in other key pulmonary and systemic inflammatory biomarkers, as well as the incidence of PPCs. A study schema is provided in Fig. 1 and Additional file 2.

### Setting

This study is being conducted at Beth Israel Deaconess Medical Center (BIDMC) in Boston. BIDMC is a 700-bed tertiary-care hospital academically affiliated with Harvard Medical School. More than 900 open-heart procedures with CPB are performed at BIDMC each year.

### Study registration

**Fig. 2:**
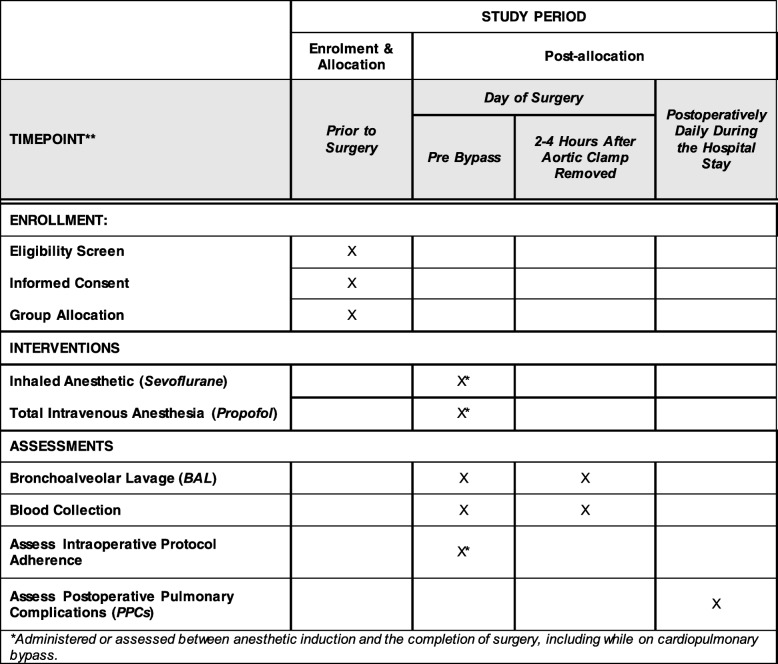
SPIRIT figure

Institutional Review Board (IRB) approval was obtained from the Committee on Clinical Investigations at BIDMC (IRB Protocol no. 2016P000306). APLICS was registered on clinicaltrials.gov with the identifier NCT02918877. Upon completion of the trial, results will be reported according to the Consolidated Standards of Reporting Trials (CONSORT) guidelines and the Standard Protocols Items: Recommendations for Interventional Trials (Fig. 2 and Additional file 1). The trial is active and ongoing; any amendments made to the protocol are reported to and approved by the BIDMC IRB before implementation.

### Inclusion and exclusion criteria

Patients are deemed eligible for enrollment if they are aged ≥ 18 years and are scheduled to undergo cardiac surgery with CPB. Patients undergoing off-pump procedures (not requiring CPB) are not included, as this study aims to evaluate the role of anesthetic type following subsequent IR lung injury following CPB. Patients are excluded if they are having emergency surgery, have a history of severe chronic obstructive or restrictive lung disease (as evident by spirometry), recent (within two weeks) use of systemic glucocorticoids, prior history of pneumothorax, allergy or contraindication to propofol, or have a personal family history, or are at increased risk of malignant hyperthermia.

### Randomization

After informed consent performed by study MDs, participants are allocated in a 1:1 fashion using block randomization to receive intraoperative anesthetic maintenance using either sevoflurane or propofol. The REDCap randomization module hosted at BIDMC is used in order to implement the study randomization schema by study personnel before any study interventions take place [13].

### Intervention group: anesthetic maintenance with sevoflurane

Patients in the sevoflurane arm receive anesthetic maintenance (between anesthetic induction and the completion of surgery, including while on CPB) with 0.7–1.5 minimum alveolar concentration (MAC) sevoflurane. As clinical conditions may dictate dosing outside of these ranges, for instance in the event of extreme variations in blood pressure, anesthesiologists are asked to maintain an average MAC dose per hour within the specified range.

### Comparison group: anesthetic maintenance with propofol

Patients randomized to the propofol arm receive anesthetic maintenance with intravenous propofol at a suggested dosing range of 50–200 μg/kg/min. Anesthesiologists are given preference as to the specific dose required to achieve a satisfactory plane of anesthesia and hemodynamic stability, as well as whether to administer additional agents typically included during a total intravenous anesthetic (e.g. remifentanyl) or to use bispectral index monitoring.

### Intraoperative clinical management in both groups

Anesthetic providers for patients in both groups are asked to avoid the administration of additional potential modulators of lung injury including nitrous oxide, steroids, and cisatracurium.

As mechanical ventilation can potentially induce confounding inflammatory lung injury and the administration of a high fraction of inspired oxygen (FiO_2_) can potentially worsen pulmonary IR injury, we have standardized the mechanical ventilation and oxygenation of the study patients according to the following guidelines:

- tidal volume will be set at 6–8 cc/kg of ideal body weight;
- positive end-expiratory pressure will be set at 2–12 cmH_2_O;
- FiO2 will be ≤ 50% and titrated to target peripheral oxygen saturation > 92%. Brief exposures (< 20 min) to a FiO_2_ of 100% for hypoxia or during the immediate post-CPB period are allowed;
- peak airway pressure goal < 40 cmH_2_O;
- plateau pressure goal < 35 cm H_2_O;
- arterial partial pressure of carbon dioxide (PaCO_2_) will be maintained at 20–60 mmHg;
- arterial partial pressure of oxygen (PaO_2_) will be maintained at 60–200 mmHg.

Protocol adherence will be facilitated by real-time conversations between the study team and the anesthesiologist; post hoc quantification of adherence will be performed by review of the anesthetic record.

### Drop-out criteria

The anesthesia team has the discretion to terminate the study protocol at any time in the interest of patient safety. This would include decisions made by the anesthesiologist to deliver a different anesthetic or to administer a steroid, for example in the event of suspected anaphylaxis. In addition, study participants will not be included in the analysis of the primary outcome if the patient is deemed at high risk for complications during a study bronchoscopy and BAL samples cannot be obtained. This includes instances of severe hypoxia (defined as a PaO_2_/FiO_2_ ratio < 100 or oxygen saturation [SpO_2_] < 90%), positive end-expiratory pressure values > 15 cmH_2_O, or patients with fraction of inspired oxygen (FiO_2_) > 80% in order to maintain a SpO_2_ 90%. Finally, the study intervention can be terminated at any time due to significant nursing or treating physician concern.

### Blinding

This is an unblinded trial. The primary outcome of the study relies on the measurement of laboratory values which are not influenced by provider bias. Furthermore, effective blinding of the anesthesia team or the study group members to randomization assignment would require the use of both a lipid emulsion placebo to mimic the administration of propofol and a sham vaporizer to mimic the administration of sevoflurane. Additionally, study members who assess secondary outcomes are limited to the identification of PPCs via chart review as documented by the treating clinical team, laboratory values, and radiographic data as interpreted by independent radiologists uninformed of study participation or group assignment.

### Study procedures: sample collection

BAL and serum collection take place at two clearly defined time points. The first sample collection occurs after anesthetic induction with endotracheal intubation and before surgical incision. The second sample collection takes place 2–4 h after removal of the aortic cross clamp. This window was chosen as it historically has been described as the time when the peak pulmonary inflammatory response occurs after reperfusion [4]. All BAL samples are obtained by a member of the study team with the appropriate training according to current BIDMC policy and procedure for BAL collection. Direct vision bronchoscopy is utilized for this purpose to minimize the risk of trauma from unintentional misplacement of a catheter. A flexible bronchoscope is passed through the endotracheal tube and gently wedged into the right lower lobe bronchus. A total of 60 cc of a sterile saline solution, typically in 30-cc instillations, is then used to lavage the cavity. Continuous suction is then used to obtain the residual lavage specimen (usually 10–20 cc) for laboratory analysis of the markers indicated below. Serum collection consists of one 10 cc ethylenediaminetetraacetic acid (EDTA) tube per time point and is obtained via catheters placed as part of the usual care for cardiac surgery.

### Specimen processing

Blood and BAL specimens are centrifuged at 25 °C at 2300 × g for 10 min. The plasma and buffy coat are separated from red blood cells, aliquoted into smaller cryovials, labeled and frozen at − 80 °C for subsequent batch analysis. The BAL specimen is also aliquoted, labeled, and frozen in a similar fashion. All specimens are labeled with a unique coded ID and will not contain any patient identifiers.

### Data collection

Clinical and demographic variables are collected and reported including age, body mass index, Society of Thoracic Surgeons (STS) predicted mortality score, and co-morbidities (e.g. history of myocardial infarction, congestive heart failure, diabetes, dyslipidemia, hypertension, stroke, peripheral vascular disease, and smoking). All patient data are stored anonymously in a REDCap database hosted at BIDMC. REDCap is a secure, web-based application designed to support data capture for clinical trials that allows customized data-collection fields to support individual trial needs. Members of the research team are responsible for building and maintaining the electronic case report form, as well as monitoring data entry for completeness, timeliness, and accuracy. During the informed consent process, individuals are asked separately to consent to specimen storage for use in future studies.

### Primary outcome: degree of inflammatory lung injury

The degree of inflammatory lung injury will be assessed by comparing the levels of key inflammatory mediators and biomarkers of lung injury found in patient BAL fluid and serum before and after exposure to cardiopulmonary bypass. Although to date there has not been a clear consensus on the ideal biomarker of pulmonary injury, we have chosen TNFα as our primary outcome to be consistent with the findings of studies done in thoracic surgery where the exposure to pulmonary IR injury is similar to cardiac surgery [8]. In addition to TNFα, the indicators tested for as secondary outcomes in this study will likely include interleukin (IL) 1b/6/8, monocyte chemoattractant protein (MCP) 1, total protein, neutrophil count, soluble receptor for advanced glycosylation end products (sRAGE), angiopoietin 1 and 2, surfactant protein D, and soluble intercellular adhesion molecule 1 (ICAM1). These indicators have been implicated as markers of lung inflammation and injury in previous studies [14]. Our list of indicators will potentially be expanded as additional novel biomarkers that are currently unknown but available at the time of analysis are identified.

### Secondary outcome: incidence of postoperative pulmonary complications

The incidence of pulmonary complications will be observed between both groups until discharge. PPCs are defined as a composite of: ARDS according to the Berlin Criteria, atelectasis, pleural effusion, pneumonia, pneumothorax, bronchospasm, exacerbation of chronic lung disease, reintubation, or ventilator dependence > 48 h [15]. This definition is in accordance with the recent consensus definition of PPCs reached by the ESA/ESICM taskforce and ARISCAT risk scoring system [16, 17]. In addition, the incidence of hypoxia (PaO_2_/FiO_2_ < 300) and respiratory acidosis (partial pressure of carbon dioxide > 45 mmHg) will be included in a separate composite model, in light of the potential relation of hypoxia and hypercarbia to lung injury and the clinical consequences of these findings with regards to patient management.

### Reporting of compliance and adverse events

A specialist within the research group will monitor protocol compliance, occurrence, and reporting of adverse events to the IRB.

### Sample size and power

A previous trial evaluating the effect of sevoflurane versus propofol during thoracic surgery found a 40% relative reduction in post-injury increase of alveolar TNFα for the sevoflurane group [8]. Based on this effect size estimate, and assuming a two-sided α of 0.05 and 80% power, we estimate that 32 participants would be required to detect a difference of > 40% in alveolar TNFα concentrations between groups. We aim to ultimately enroll a total of 20 individuals per group in order to assess potential differences in other inflammatory markers as secondary outcomes. Additional participants may be enrolled in order to ensure a total of 40 analyzable datasets are obtained after accounting for potential withdrawal of patients before randomization (e.g. surgery changes, patient withdrawal). Analysis of our secondary aims will likely be underpowered but will be used to identify the incidence of PPCs and support future power calculations into the relationship between anesthetic use and PPCs.

### Statistical analysis

We will use SAS software version 9.4 or later (SAS Institute, Cary, NC, USA) to conduct all analyses. Descriptive statistics of the data will be assessed and presented as mean (± standard deviation), median (interquartile range), or frequencies and proportions depending on variable type and distribution. Normality will be assessed with the use of the Shapiro-Wilk test. Differences between groups in continuous variables will be compared using parametric or non-parametric t-tests as appropriate. Categorical data will be compared using a Chi-square or Fisher’s exact test for small cell counts. Two-sided *p* values < 0.05 will be considered statistically significant for all analyses.

### Analysis of the primary outcome

Our primary outcome, differences in BAL TNFα levels found before and after exposure to CPB, will be assessed using a paired t-test or Wilcoxon signed rank sum test as appropriate. A similar analysis will be performed for additional BAL and serum biomarkers as secondary outcomes. It is anticipated that randomization should account for potential differences between groups at baseline; however, more sophisticated adjustment, for example with multivariable logistic regression, for potential confounders may occur if differences between groups persist after randomization in relevant categories such as surgery type and duration of aortic cross clamping.

### Analysis of secondary outcomes: postoperative pulmonary complications

In order to assess the relationship between anesthetic type and PPCs, logistic regression will be used, with data presented as odds ratios and 95% confidence intervals. In the event that the outcome is more common than expected (> 10% incidence), we will employ the use of log-binomial regression, presenting relative risk estimates and their confidence intervals. Individual outcomes will also be reported to see if any early trends emerge between the sevoflurane and propofol groups. In addition, we plan to perform two subgroup analyses of PPCs, one stratified by baseline risk and the other including the variables for hypoxia and hypercarbia. Risk will be calculated according to the ARISCAT preoperative pulmonary risk score and assessed using quartiles in order to reduce the potential for results to be skewed by the distribution of the data. Components of the ARISCAT scores will be calculated for each patient based off their ASA classification, functional status, and presence of preoperative sepsis. Similar regression analyses will then be used to ascertain differences between anesthetic groups and incidence of PPCs in each strata of risk.

### Protocol funding sources and their role

This study is supported by the American Society of Anesthesiologists’ Foundation for Anesthesia Education and Research. Funds have been allotted from this organization to support principal investigator time and effort. The scientific content of the study protocol and execution of the trial is in no way influenced by this funding source. Protocol development, execution, and adherence, as well as scientific content development are supported under the Center for Anesthesia Research Excellence (CARE) within the Department of Anesthesia, Critical Care and Pain Medicine at BIDMC.

## Discussion

The APLICS trial will be the first to evaluate the potential for anesthetic choice to reduce inflammatory lung injury after cardiac surgery. Given the high incidence of PPCs in this cohort, a reduction in inflammation could theoretically lead to an improvement in outcomes for an increasingly large group of at-risk patients. Recently, a similar trial conducted in lung cancer surgery patients demonstrated a significant reduction in PPC incidence from the use of sevoflurane compared to propofol [18]. The proposed mechanism for this finding was a decrease in pulmonary inflammation as evident by a reduction in the release of pulmonary pro-inflammatory cytokines in the sevoflurane group. A similar result from our trial would add to the growing body of evidence that the use of the inhaled anesthetics including sevoflurane for surgical anesthesia can lead to a reduction in pulmonary inflammation and potentially improved clinical outcomes.

Our study has several limitations. First, APLICS is not powered to detect a difference in clinical outcomes. The reasons for this design choice are multifactorial. Although the mechanism for lung injury in thoracic and cardiac surgery are similar, a reduction in lung inflammation with the use of the inhaled anesthetics compared to propofol has not yet been demonstrated in this patient group. Therefore we thought it important to power a smaller study to detect a difference in chemical inflammatory mediators before conducting a larger clinical trial aimed at identifying potential differences in clinical outcomes. A second limitation of APLICS is the potential for bias that comes with the lack of assessor blinding. As discussed above, the requirements for the adequate intraoperative blinding of the clinical and study teams to the group assignment were thought to be excessive given the objective nature of the primary outcome. A more relevant source of bias through lack of blinding can occur during the assessment of the secondary outcome, PPCs. Our group has taken several steps to reduce this potential source of bias. First, our definition of PPCs is consistent with international consensus. Second, the determination of PPCs is largely made through the interpretation of laboratory results and radiographic data obtained through the course of usual care and thus interpreted by clinical personnel unaware of the study and group assignment. In the cases where there is potential for a subjective assignment of PPCs, for example pneumonia, study staff are restricted to indentifying pneumonia only from chart review of diagnoses made by clinical staff who were unaffiliated with the trial.

Another limitation of our study is the potential for propofol exposure in the sevoflurane group. Clinicans are permited to use any anesthetic induction agent of their choosing, including propofol. Since we hypothesize that the critical exposure period for the anesthetic-mediated prevention of IR lung injury is during CPB, this potential for contamination between groups was thought to be minimal in comparison to the safety benefit afforded to the clinical team in achieving a safe anesthetic induction in patients at high risk for hemodynamic compromise. Along the same lines, anesthesia and ICU providers were also allowed freedom of choice in deciding the safest and most effective means of postoperative sedation, including the use of propofol infusions. The scientific benefit of maintaining strict control of the study exposure is outweighed by a need to maximize patient safety.

The APLICS trial will be the first to evaluate whether the use of sevoflurane compared to propofol for anesthetic maintenance can reduce the degree of inflammatory lung injury following exposure to CBP. A positive result from APLICS would add to a growing body of evidence supporting the potential lung protective effects of the volatile anesthetics. If such a finding could potentially lead to a reduction in patient morbidity by limiting PPCs, it could mean that the simple intervention of anesthetic choice could impact the successful recovery of thousands of cardiac surgical patients per year.

## Additional files

Additional file 1: SPIRIT checklist. (DOC 123 kb)

Additional file 2: WHO Trial Registration Data Set - Structured Summary. (DOCX 14 kb)

## Data Availability

Data from the current study is available from the corresponding author upon reasonable request.

## Abbreviations

APLICS: Anesthesia to Prevent Lung Injury in Cardiac Surgery
ARDS: Acute respiratory distress syndrome
ARISCAT: Assess Respiratory Risk in Surgical Patients in Catalonia
BAL: Bronchoalveolar lavage
BIDMC: Beth Israel Deaconess Medical Center
CARE: Center for Anesthesia Research Excellence
cc: Cubic centimeter
CONSORT: Consolidated Standards of Reporting Trials
CPB: Cardiopulmonary bypass
ESA: European Society of Anaesthesiology
ESICM: European Society of Intensive Care Medicine
FiO_2_: Fraction of inspired oxygen
H_2_O: Water
ICAM: Intercellular adhesion molecule
ICU: Intensive care unit
IL: Interleukin
IRB: Institutional Review Board
Kg: Kilogram
MAC: Minimum alveolar concentration
MCP: Monocyte chemoattractant protein
PaCO_2_: Arterial partial pressure of carbon dioxide
PaO2: Arterial partial pressure of oxygen
PPCs: Postoperative pulmonary complications
REDCap: Research Electronic Data Capture
SPIRIT: Standard Protocol Items: Recommendations for Interventional Trials
SpO_2_: Peripheral oxygen saturation
sRAGE: Soluble receptor for advanced glycosylation end-products
STS: Society of Thoracic Surgeons
TNFα: Tumor necrosis factor alpha
