# Supplementary material for: Anesthetics to Prevent Lung Injury in Cardiac Surgery (APLICS): a protocol for a randomized controlled trial
Source: Trials. 2019 May 31;20:312. doi: 10.1186/s13063-019-3400-x (PMC6544964; doi:10.1186/s13063-019-3400-x)
Supplement: Supplementary file 2 — WHO Trial Registration Data Set - Structured Summary. (DOCX 14 kb) [file 13063_2019_3400_MOESM2_ESM.docx]

Supplemental File: WHO Trial Registration Data Set- Structured Summary

| Data Category | Information |
| --- | --- |
| Primary registry, trial identifying number | Clinicaltrials.gov Identifier - NCT02918877 |
| Date of registration in primary registry | September 29, 2016 |
| Secondary Identifying Numbers |  |
| Sources of Monetary Support | American Society of Anesthesiologists’ Foundation for Anesthesia Education and Research – Mentored Research Training Grant |
| Contact for Public queries | BO, Department of Anesthesia, Beth Israel Deaconess Medical Center, Boston MA, USA |
| Contact for Scientific queries | BO, Department of Anesthesia, Beth Israel Deaconess Medical Center, Boston MA, USA |
| Public Title | Anesthetics to Prevent Lung Injury in Cardiac Surgery |
| Scientific Title | Anesthetics to Prevent Lung Injury in Cardiac Surgery |
| Country of Recruitment | USA |
| Health Problem Under Investigation | Inflammatory lung injury after cardiac surgery |
| Key inclusion and exclusion criteria | Age ≥ 18 years, elective or urgent cardiac surgery on cardiopulmonary bypass (CPB). Exclusion for emergency surgery, history of severe obstructive or restrictive lung disease, history of steroid use within 2 weeks, personal/family history of malignant hyperthermia, risk of malignant hyperthermia |
| Study type | Interventional  Allocation: randomized  Interventional model:  Masking: unblinded |
| Date of First Enrollment | June 2017 |
| Target Sample Size | 45 |
| Recruitment Status | Active and enrolling |
| Primary Outcome | Inflammatory lung injury: BAL concentration of TNFα |
| Key Secondary Outcomes | Incidence of postoperative pulmonary complications. Other serum and BAL mediators of inflammatory lung injury. |
